# Nanoparticle Layer Deposition for Plasmonic Tuning of Microstructured Optical Fibers

**DOI:** 10.1002/smll.201001071

**Published:** 2010-10-19

**Authors:** Andrea Csaki, Franka Jahn, Ines Latka, Thomas Henkel, Daniell Malsch, Thomas Schneider, Kerstin Schröder, Kay Schuster, Anka Schwuchow, Ron Spittel, David Zopf, Wolfgang Fritzsche

**Affiliations:** Institute of Photonic Technology (IPHT)PO Box 100 239, 07702 Jena, Germany

**Keywords:** localized surface plasmon resonance, metal nanoparticles, microstructured optical fibers, plasmonic layers, sensors

## Abstract

Plasmonic nanoparticles with spectral properties in the UV-to-near-IR range have a large potential for the development of innovative optical devices. Similarly, microstructured optical fibers (MOFs) represent a promising platform technology for fully integrated, next-generation plasmonic devices; therefore, the combination of MOFs and plasmonic nanoparticles would open the way for novel applications, especially in sensing applications. In this Full Paper, a cost-effective, innovative nanoparticle layer deposition (NLD) technique is demonstrated for the preparation of well-defined plasmonic layers of selected particles inside the channels of MOFs. This dynamic chemical deposition method utilizes a combination of microfluidics and self-assembled monolayer (SAM) techniques, leading to a longitudinal homogeneous particle density as long as several meters. By using particles with predefined plasmonic properties, such as the resonance wavelength, fibers with particle-adequate spectral characteristics can be prepared. The application of such fibers for refractive-index sensing yields a sensitivity of about 78 nm per refractive index unit (RIU). These novel, plasmonically tuned optical fibers with freely selected, application-tailored optical properties present extensive possibilities for applications in localized surface plasmon resonance (LSPR) sensing.

## 1. Introduction

Noble metal nanoparticles show distinguished optical properties due to resonant behavior based on the density oscillations of their conductive electrons. These oscillations excite the so-called particle plasmon polaritons with defined localized surface plasmon resonance (LSPR).^1^, ^2^ The position of the LSPR strongly depends on the material properties,^3^, ^4^ composition (e.g., alloy^5^ or core–shell^6^, ^7^), dimension, and shape of the particles. These factors can be adjusted by chemical synthesis. Using colloidal synthesis, gold, silver, copper, platinum, and palladium nanoparticles can be prepared in the shape of spheres,^8–10^ triangles,^11^, ^12^ nanorods,^8^, ^9^ and other geometries.^9^^–^11 However, since the LSPR effect is an interface phenomenon, not only the particle properties but also the immediate surroundings determine the optical behavior. Plasmon particles show a large spectral response to changes in the surrounding media, for example, by binding analyte molecules onto the particle surface using (bio)affinity interactions between capture and probe,^12^, ^13^ which indicates their potential for LSPR sensing.^12^ Different kinds of plasmon particles have varying levels of sensitivity. Particles with anisotropic geometries and core–shell particles offer higher sensitivity compared to spheres and homometallic particles, respectively.^14^ Plasmon particles can act as transducer structures in the form of single particles,^15^, ^16^ (sub-)mono layers,^17^ solutions,^18^ or complex nanostructures.^19^ Additionally, the particles can induce local field enhancement for other sensoric principles, like surface-enhanced Raman spectroscopy (SERS),^20^, ^21^ as well as for enhanced luminescence or fluorescence.^22^, ^23^ In this Full Paper, we present the use of NLD to prepare defined plasmon layers from selected particles with tailored plasmon resonances inside optical fiber structures for the development of novel LSPR sensing devices.

**Scheme 1:**
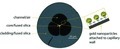
A front view of a SCF, a special kind of MOF, with a scheme of the proposed inner coating with plasmonic nanoparticles using NLD.

Microstructured optical fibers (MOFs) hold such promise for the creation of new optical devices that they are the subject of intensive study in the scientific community.^29^, ^30^ Such fibers exhibit various arrangements of air holes and, by choosing an appropriate structure, the spectral and spatial characteristics of the guided light can be engineered. One type of MOF is the suspended core fiber (SCF). SCFs consist of a solid central core surrounded by an arrangement of 2–6 air holes, which run longitudinally along the length of the fiber, and with the core suspended on thin bridges (see Scheme 1).^24^ The light is guided by the effective index contrast between the massive core and the surrounding air holes. It has been proposed to harness the evanescent field of the guided light for the sensing of gases, liquids, and analytes surrounding the core (within the holes of the fiber).^25^ In general, such fibers offer a high sensitivity due to their potential use for long interaction lengths.

Recently, there has been great interest in fibers with incorporated metallic thin films or nanoparticles in order to bring about the next generation of photonic or plasmonic devices. The incorporation of metals into optical-fiber geometry allows the guiding of the photon transport within the active plasmonic region, yielding highly integrated devices with unique excitation and detection geometries.^26–29^ In recent years, high-pressure chemical deposition techniques, for example, chemical vapor deposition (CVD), have been developed to include a wide range of optically important materials within the MOF capillaries.^29^, ^30^ One such integration was the high-pressure deposition of silver nanoparticles, which allowed the development of a fiber-optic SERS sensor.^31^, ^32^ Besides the high pressure, 10–100 MPa, an additional heat treatment with a temperature of 200 °C is required, which can have adverse effects on the mechanical stability of the fiber by damaging the standard outer polymer coating. The metal layer can be realized only for lengths of 15 cm with an approximately 50-μm channel diameter and only after a 2-h incubation period. In addition, such a particle coating is only homogeneous for 5–6 cm in the middle section of the MOF since covering gradients were induced by depletion of particles. Therefore, when capillary filling is utilized for SERS applications, only the front end is sufficiently coated.^33^ This method can be performed at 60 °C. A mixture of analyte and nanoparticles were used for SERS in MOFs.^34^ The capillaries of 40-cm-long pieces of MOF were coated by in situ synthesis of silver particles from dextrose and silver nitrate in static deposition procedures.^35^ Such coating shows a relatively rough and granular surface and the layer thickness cannot be adjusted exactly. Homogeneous silver layers can be produced by vigorous shaking during the deposition;^36^ however, such shaking is only possible for short fiber pieces.

In general, deposition methods for long MOFs must necessarily be performed at room temperature with homogeneous particle coverage and adjustable covering density. We introduce here a dynamic low-pressure chemical deposition of metal nanoparticles, which are attached to a self-assembled adhesive monolayer on the inner surfaces of the MOF. This so-called NLD technique is based on the self-assembled monolayer (SAM) techniques^37^, ^38^ for oxide surfaces using silane chemistry and controlled microfluidic management, with a microstructured fluid chip for the covering procedure, and can be employed with various types of metal nanoparticles as layer components.

## 2. Nanoparticle Layer Deposition in Holes of MOFs

**Figure 1:**
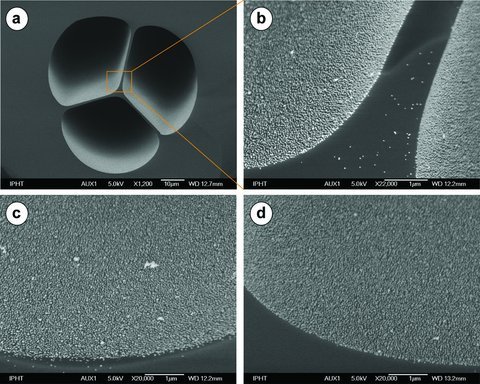
SEM images of the inner walls of the MOFs coated with gold particles (30-nm-diameter spheres): a) An overview and b) zoomed in. c) Tilted front view of one hole’s cross section at the starting point. d) A view of the fiber end. A homogeneous particle density – independent of curvature – is apparent.

The preparation of nanoparticle-based plasmonic structures on the internal capillary walls of MOFs was realized using NLD. This technique combines SAM techniques, microfluidic control of the surface chemistry, and guided particle deposition. Microfluidic chips were designed for optimally interfacing the MOFs. The preferred MOFs, such as the SCFs, were coupled into the microfluidic chip and the capillary walls were chemically modified by the perfusion of the silanes. The resulting functional layer was a chemical adhesive for metal nanoparticles due to its amino modification,^39^ as displayed in Scheme 1. Metal nanoparticles were prepared in different shapes, dimensions, and with different materials and the selected particle solutions were incubated by continuous flow. The incubation time was <1 h for 40-cm-long fiber pieces and ∼60 h for 6-m-long pieces; for the latter, only 4 mL of particle solution was used. A successive inner saturation of the MOF with nanoparticles can be observed over the incubation time: the color front migrated along the fiber with a speed of ∼2 cm min^−1^. The coating uniformity that resulted, that is, the nanoparticle density and the thickness of our layers, was constant over tens of centimetres up to 6 m. A homogeneous coating density was observed independently on the local curvature of the capillary-channel cross section (**Figure** **1**a,b). Scanning electron microscopy (SEM) images clearly show particle (sub)monolayers at saturation coverage (Figure 1c,d). A density of ∼450 particles μm^−2^ for 30-nm gold nanoparticles was determined in both the start and end region. Compared to other coating techniques with gelatine precursor layers for 35-nm silver particles (density of 1 particle μm^−2^),^40^ the presented dynamic deposition technique offers a significantly higher particle density.

The method presented for the modification of MOFs/SCFs was shown as a defined coating technique of the capillaries using a fluidic chip, the respective fluidic periphery, and the chemical deposition technique based on SAM techniques. The resulting layer thickness is adjustable by changing the selected particle dimension. The next section focuses on the optical characterization of plasmonically tuned SCF fibers prepared by the method discussed.

## 3. Optical Properties of the Plasmonically Tuned SCF Fibers

The optical properties of the resulting, internally coated MOFs (SCFs) are identical to the properties of the employed colloidal suspensions of plasmon particles, as shown in **Figure** **2**. The optical behavior was adjusted by selecting plasmon particles that absorb in the UV range (Pt and Ag spheres), visible range (Au spheres and Ag triangles), or near-infrared range (Ag triangles and Au nanorods).

**2:**
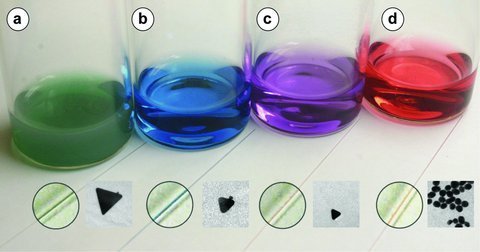
Colloidal nanoparticles in solution and as the internal layer in SCFs. a) Silver triangles with ∼120-nm edge length. b) Silver triangles of ∼50-nm edge length. c) Silver triangles of ∼26-nm edge length. d) Gold spheres, 30 nm in diameter. Transmission electron microscopy (TEM) insets are 200 nm × 200 nm.

**Figure 3:**
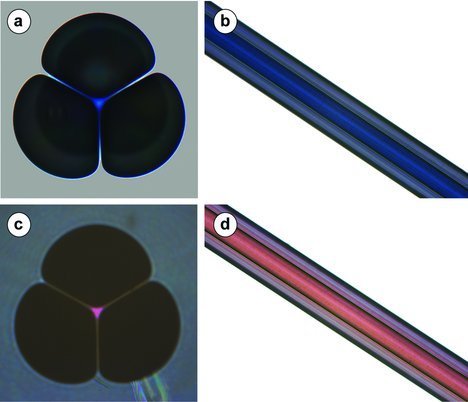
Two types of plasmonically tailored SCFs: end faces and side views. Blue and red colors result from the utilized nanoparticles: a,b) blue – Ag triangles with ∼50-nm edge length; c,d) red – Au spheres 30 nm in diameter are visible both in the SCF core (a,c) as well as in the channels (b,d).

As shown in **Figure** **3**, a successful inner coating with plasmon particles in the visible spectral range can be easily confirmed by microscopic inspection or even by the naked eye, either from the end face (Figure 3a,c) or from the side (Figure 3b,d). The plasmonically tailored fibers showed measurable transmission only for short fiber pieces of ∼3-mm long. The high losses are clearly explainable by the high particle coverage realized, which causes a very strong interaction of the relatively small core with the extremely high number of nanoparticles. By comparison, the 1 particle μm^−2^ coverage described in Oo et al.^40^ resulted in a loss of 0.57 dB m^−1^ and the particle density of the presented dynamic deposition technique is ∼450 times higher. Therefore, the expected attenuation should be approximately three orders of magnitude higher. Changing the particle surface density directly influences the efficiency and the length needed to make such a sensor useful. High particle coverage allows for dissection of the plasmonically tuned long fiber on a short (mm) length scale and therefore sensoric applicable segments. So, the cost-effective preparation of novel miniaturized sensor devices is possible. Otherwise, for the utilization of long interaction length in MOFs, the density of gold particles on the inner surfaces has to be decreased. The use of a mixed monolayer in the NLD process enables the control of the particle density and thereby the adjustment of the attenuation. Investigations for such a control of the particle coverage are in progress.

**Figure 4:**
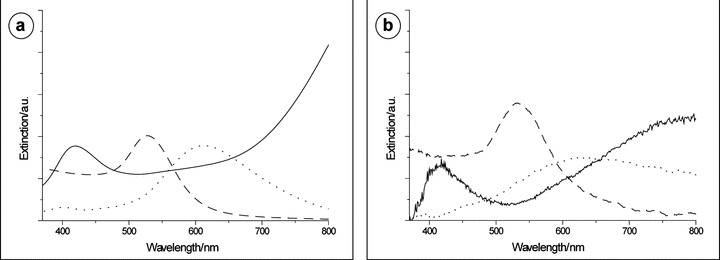
Extinction spectra of the a) utilized nanoparticle solutions and b) the correlated plasmonically tuned SCFs. Dotted lines: silver triangles with ∼120-nm edge length. Dashed lines: silver triangles of ∼50-nm edge length. Solid lines: gold spheres 30 nm in diameter.

Transmission measurements of the MOFs are needed in order to utilize the plasmonic particle layer as a transducer for sensing changes in the refractive index. The transmission spectrum of a fiber will usually be measured longitudinally, that is, in the fiber axis along the fiber core. However, in the case of plasmonically tuned SCFs with saturated particle coverage, a longitudinal transmission measurement was not possible due to the strong attenuation already mentioned. Therefore, a transversal measurement setup was preferred, by which illumination as well as collection of light transversally to the fiber axis occurred. The resulting effective interaction length was about four times the thickness of the nanoparticle layer, which proved to be sufficient for transmission measurements on a SCF coated with particles at high surface density. The fiber piece to be measured was positioned vertically in the collimated beam of the white-light source. Directly behind the SCF, a large core fiber selected only that part of the light that was transmitted through the SCF. A spectrum of a SCF without inner coating was used as the reference to calculate an extinction spectrum from the transmissions. In **Figure** **4**, an extinction spectrum of plasmonic particle solutions and SCFs coated with correlated particles are compared. The extinction peaks of certain nanoparticles (30-nm gold spheres, silver triangles with ∼120-nm, ∼50-nm, and ∼26-nm edge lengths) are well separated spectrally. The extinction spectrum measured from nanoparticles in solution (Figure 4a) can also be reproduced with the inner-coated SCF (Figure 4b).

The graphs for the 30-nm gold spheres (dashed lines in Figure 4) are nearly identical. For the silver triangles with ∼50-m edge length (dotted lines in Figure 4) and the silver triangles with ∼120-nm edge length (solid lines in Figure 4), only the peak positions in both systems fit well, although the peak widened when the particles were deposited inside the glass fiber. This is the result of a substrate effect, as the particles in colloidal solutions are surrounded only by water. In SCFs, the particles are partially surrounded with the fiber material, silica glass. This induces a different proportional refractive index in the medium around the particles and effects small shifts in spectra and broader peaks of the larger and nonspherical particles.^1^, ^41^ In addition, dipole–dipole interactions between the particles in the plasmonic layer are possible.^42^

Transmission measurements have shown that layers of plasmonic particles from gold seem to be stable in the fiber over several months. SCFs with a layer of 30-nm gold spheres turned out to be very stable in spite of the fill and refill processes. The measured transmission curves were reproducible for more than five refill cycles. In addition, the testing of the same plasmonically tuned SCF in different positions shows that not only are their SEM images very similar but so are their spectral properties.

**Figure 5:**
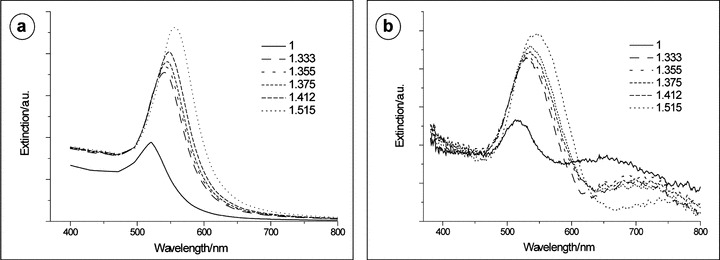
a) Theoretical calculations and b) the real measurements for the refractive-index changes for a plasmonically tuned SCF with 30-nm gold spheres.

In order to characterize the optical properties, SCFs coated with 30-nm gold particles were tested as the sensor. The sensitivity was determined by transversal measurement with solutions of different refractive index, which were injected into fiber channels with the same fluidic setup as for the particle-layer preparation. For the 30-nm gold spheres, the theoretical calculations show a strong dependence on the refractive index (**Figure** **5**a). Experimental measurements (Figure 5b) with plasmonically tuned SCFs confirmed these theoretical simulations. The difference in peak width and the subsidiary peak was induced by the substrate effect for plasmonic particle layers on planar glass surfaces and by the nonspherical geometry of the real particle samples.^1^, ^41^, ^43^, ^44^ The sensitivity of 30-nm-gold-particle-modified SCF was determined to be ∼78 nm per refractive index unit (RIU). This value compares well to the sensitivity for LSPR sensing with gold nanospheres, ∼72 nm RIU^−1^, in so-called *nanoSPR*.^17^ By proper selection of plasmon particles for plasmonic tuning of SCFs, an increased sensitivity of ∼600 nm RIU^−1^ can be achieved.^14^

## 4. Conclusion

We have demonstrated a novel method for the generation of plasmonic nanoparticle layers inside the channels of microstructured optical fibers, especially for SCFs, based on SAM techniques. By using microchips for the fluidic coupling, a reproducible, cost-effective, and contamination-free nanoparticle deposition is possible. With the presented method, nanoparticles in a great variety of materials, shapes, and sizes can be used for the deposition. Optical characterization, as well as electron microscopic evaluation, confirmed the even deposition of the holes and the constant population density over fiber lengths of several meters. The possibility of preparing fibers with plasmonic properties in the UV-to-near-infrared spectral range then exists. A transversal measurement setup can be utilized because of the high population density, allowing the ports for optical and fluidic coupling to be separated. This enables the principle testing of the plasmonic tuned fiber system for sensing applications. For detection and measurements of a liquid analyte, the filling setup tested in the coating procedure described above can be applied. This technique allows the complete filling of a piece of a SCF without bubbles remaining and the capillaries of the same piece of SCF are still easy to repeatedly clean, refill, and dry. In a proof-of-principle experiment, the refractive-index-dependent shift of the LSPR peak was demonstrated. For particle layers with 30-nm gold spheres in MOFs, a sensitivity of ∼78 nm RIU^−1^ was measured. The presented system offers a vast potential for the development of innovative sensors based on LSPR and local field enhancement, like SERS or enhanced fluorescence. The fiber channels can be used both for the transport of analyte molecules as well as for the generation of the sensor signal on the particle-based plasmonic transducer layer. The actual transmission losses, and with this the usable fiber length, could be tuned with an adapted particle density. Experiments concerning adjustable particle density are in progress. This method provides a miniaturized, cost-effective sensor for bioanalytical and diagnostic applications.

## 5. Experimental Section

*Preparation of Microfluidic Chips and* *Fluid-Coupling*: Microfluidic chips for interfacing the MOFs/SCFs were prepared by wet etching and anodic bonding of two glass substrates using a silicon-bond support layer.^45^ In brief, fluid and fiber port channels were etched into two glass substrates, with an etch depth of 65 μm. After the bonding of two half-channels, a total height of 130 μm was realized, which was optimally suited for the interfacing of optical fibers with an outer diameter of 125 μm. The SCFs were prepared using high silica glass capillaries by “stack-and-draw” technology.^46^ Their outer diameter was 125 μm, the core diameter was 3.2 μm, and the dimension of the holes was 30 × 40 μm with 0.9-μm-thick bridges.^47^

SCFs with the protective plastic coating (acrylate) were inserted into the microfluidic chip and glued into the fluidic output. The chip was then connected to a syringe pump system (neMESYS, cetoni GmbH, Korbussen, Germany) by Teflon tubes (Jasco, Gross-Umstadt, Germany).

*NLD Process*: Chemical activation solution, washing solutions, and silane were incubated by continuous flow. All chemicals were purchased from Sigma-Aldrich (Taufkirchen, Germany, puriss pro analysi). Three-times-distilled (3d) water and solutions were filtered through 0.22-μm pore filters before use (Millipore, Schwalbach, Germany). Metal nanoparticles were prepared in different shapes, dimensions, and with different materials by colloidal synthesis: gold spheres^48^, ^49^ and nanorods,^50^ silver triangles,^51^ and platinum spheres.^52^ Transmission electron microscopy (TEM) measurements (Zeiss CEM 902A, Jena, Germany) were used to characterize the resulting particle’s dimension and shape, respectively. Particle solutions (≍10^11^ particles μm^−2^) were incubated by continuous flow with 1–8 μL min^−1^ at room temperature.

*Characterization of the Plasmonic Particle Layer in MOFs*: The coating uniformity was characterized for cleaved fiber pieces from different positions along the inner-coated SCFs by SEM measurements (Zeiss DSM 960, Jena, Germany). For the spectral characterization of the plasmonically tuned fibers, a white-light source (Mikropack DH2000, OceanOptics, Duiven, Netherlands) and fiber spectrometer (Spectro 320D, Instrument Systems GmbH, Munich, Germany) were employed. Microscopic images were taken with an AxioImager*,* equipped with a color camera (AxioCam mrc5, Carl Zeiss, Jena, Germany) in transmission and reflection mode.
